# The prognostic value of peritoneal metastases in patients with gastric cancer: a nationwide population-based study

**DOI:** 10.1016/j.eclinm.2025.103109

**Published:** 2025-02-13

**Authors:** Niels A.D. Guchelaar, Micha J. de Neijs, Bo J. Noordman, Heilida E.C. Graaf, Irene E.G. van Hellemond, Pieter C. van der Sluis, Esther Oomen-de Hoop, Sjoerd M. Lagarde, Rob H.A. Verhoeven, Stijn L.W. Koolen, Misha D.P. Luyer, Ignace H.J.T. de Hingh, Hanneke W.M. van Laarhoven, Bianca Mostert, Bas P.L. Wijnhoven, Ron H.J. Mathijssen

**Affiliations:** a Department of Medical Oncology, Erasmus MC Cancer Institute, University Medical Centre Rotterdam, Rotterdam, the Netherlands; b Department of Surgery, Division of Surgical Oncology and Gastrointestinal Surgery, Erasmus MC Cancer Institute, Rotterdam, the Netherlands; c Department of Medical Oncology, Catharina Cancer Institute, Eindhoven, the Netherlands; d Department of Research & Development, Netherlands Comprehensive Cancer Organization (IKNL), Utrecht, the Netherlands; e Cancer Centre Amsterdam, Cancer Treatment and Quality of Life, Amsterdam, the Netherlands; f Department of Medical Oncology, Amsterdam UMC Location University of Amsterdam, Amsterdam, the Netherlands; g Department of Pharmacy, Erasmus Medical Centre, Rotterdam, the Netherlands; h Department of Surgery, Catharina Cancer Institute, Eindhoven, the Netherlands; i Department of Epidemiology, GROW-School for Oncology and Developmental Biology, Maastricht University, Maastricht, the Netherlands

**Keywords:** Gastric cancer, Peritoneal metastases, Prognosis, Metastases, Chemotherapy

## Abstract

**Background:**

The peritoneum is a common metastatic site in gastric cancer. The prognosis of synchronous peritoneal metastases compared to other metastatic sites in gastric cancer remains understudied. This study aims to evaluate the impact of peritoneal metastases on survival in patients with metastatic gastric cancer.

**Methods:**

Patients with gastric cancer and synchronous metastases between 2015 and 2020 were identified from the nationwide Netherlands Cancer Registry. Patients were categorized based on the site of metastases. Median overall survival (OS) was calculated for each metastatic site group. Multivariable Cox regression analyses were performed to evaluate the association between patient, tumour, and treatment characteristics, including the impact of systemic therapy, on OS.

**Findings:**

A total of 4072 patients were included, of whom 1835 (45.1%) had peritoneal metastases. Of these, 58.1% had isolated peritoneal metastases. For patients with metastatic gastric cancer treated with systemic therapy, the median OS was 9.0 months (95% confidence interval (CI): 8.6–9.5), compared to 1.7 months (95% CI: 1.7–1.9) for treatment-naïve patients, who received only palliative care. The survival for patients with isolated peritoneal metastases (4.4 months, 95% CI: 4.0–4.8 months) was similar to those with isolated non-peritoneal metastases (4.6 months, 95% CI: 4.2–5.1 months, adjusted HR: 0.94, 95% CI: 0.86–1.03, p = 0.185). Systemic therapy was associated with comparable survival in patients with peritoneal metastases and those with metastases at other sites.

**Interpretation:**

This study demonstrates that there is no statistically significant difference in survival between patients with isolated peritoneal metastases and those with isolated non-peritoneal metastases in gastric cancer. Our findings emphasize the unique prognostic landscape for peritoneal metastases in gastric cancer, underscoring the need for disease-specific evaluations, rather than relying on assumptions derived from other cancer types.

**Funding:**

None.

**Research in context**

**Evidence before this study**

We searched PubMed with no language restrictions for articles published up to July 1, 2024, using the search terms “gastric cancer”, “peritoneal”, “metastases”, and “prognosis”. This literature search identified four relevant studies, all of which were limited by relatively small sample sizes, with cohorts of up to 500 patients. Two of these studies found that the presence of peritoneal metastases in gastric cancer was associated with worse survival outcomes. However, the other two studies did not find evidence supporting a negative prognostic value of peritoneal metastases.

**Added value of this study**

The present study provides a large-scale, population-based analysis of the prognostic value of peritoneal metastases in patients with metastatic gastric cancer. By utilizing data from the nationwide Netherlands Cancer Registry, this study offers an evaluation of survival outcomes based on metastatic site in a relatively large sample size, distinguishing between isolated peritoneal and isolated non-peritoneal metastases. Unlike previous studies that were limited by small sample sizes, this research shows that there is no statistically significant difference in overall survival between patients with isolated peritoneal metastases and those with isolated non-peritoneal metastases in gastric cancer.

**Implications of all the available evidence**

Our findings suggest that the prognostic value of peritoneal metastases on survival in gastric cancer differs significantly from the well-established negative prognostic effect seen in other cancer types. This underscores the need for further investigation into the biological mechanisms underlying site-specific metastases in gastric cancer and their potential role in guiding treatment strategies.

## Introduction

Gastric cancer frequently presents at an advanced stage, with approximately 40% of patients being diagnosed with metastatic disease at the time of diagnosis.^1^^,^^2^ The peritoneal cavity is a significant site of dissemination. Recent population-based studies estimate the incidence of peritoneal metastases at diagnosis to range from 10% to 21%.^3^ Other common metastatic sites for gastric cancer include the liver (12%), distant lymph nodes (11%) and lung (2%).^4^ Progression of peritoneal metastases can lead to intestinal stenoses, dysmotility, and ascites, resulting in a range of symptoms such as food intolerance, bloating, nausea, emesis, and abdominal pain.^5^

In recent years, given the large disease burden of peritoneal metastases and the hypothesis that systemic chemotherapy is insufficiently active in the peritoneal cavity, research has focused on local treatments within the peritoneal cavity for managing peritoneal metastases of gastric origin. For patients with limited isolated peritoneal metastases (defined as a peritoneal cancer index (PCI) <7 and/or tumour positive peritoneal cytology), cytoreductive surgery combined with a single heated administration of chemotherapy (CRS-HIPEC) has emerged as a potentially effective treatment option.^6^, ^7^, ^8^ Meanwhile, for patients with more extensive peritoneal metastases, systemic chemotherapy in combination with repeated administration of cytotoxic drugs directly into the peritoneal cavity, such as catheter-based intraperitoneal chemotherapy or pressurized intraperitoneal aerosol chemotherapy (PIPAC), is emerging as a promising therapeutic strategy.^9^^,^^10^

The prognosis of patients with peritoneal metastases has been studied extensively in other cancer types, particularly in colorectal cancer. By combining data from multiple randomized trials, Franko et al. showed that patients with isolated peritoneal metastases of colorectal origin have significantly shorter overall survival than those with other isolated sites of metastases.^11^ However, the relative prognosis of peritoneal metastases in patients with gastric cancer compared to other sites of metastases is not well known, with only a few small studies available, presenting contradictory results.^12^, ^13^, ^14^, ^15^ Additionally, randomized controlled trials often underrepresent patients with peritoneal metastases due to the requirement for measurable disease and the urgent need for systemic therapy in these patients, leading to exploratory and underpowered subgroup analyses.

Therefore, the aim of this study was to investigate the survival of patients with metastatic gastric cancer and synchronous peritoneal metastases in a nationwide, daily practice setting. The objectives were to compare the overall survival and clinical characteristics of patients with synchronous metastatic gastric cancer, distinguishing between those with isolated peritoneal metastases, those with non-isolated peritoneal metastases, and those with metastases not involving the peritoneum.

## Methods

### Study design

Patients diagnosed with synchronous metastatic gastric cancer in the Netherlands from 2015 to 2020 were identified through the Netherlands Cancer Registry (NCR). Gastric cancer was defined as C16.0 to C16.9, according to the third revision of the International Classification of Diseases for Oncology (ICD-O-3), and also included tumours from the gastroesophageal junction and cardia. The NCR is a population-based registry that registers all newly diagnosed cancer cases through direct linkage with the national pathological archive and Dutch hospital data. Trained registrars gather patient, tumour, and treatment data from hospital records. Metastatic locations are determined based on imaging reports, pathological findings, and clinician documentation. Vital status updates are annually obtained via linkage with municipal administrative databases. This study was performed in accordance with the Strengthening the Reporting of Observational Studies in Epidemiology (STROBE) guidelines.^16^

In this study, only patients with synchronous metastatic gastric cancer were included. Synchronous metastases were defined as those diagnosed before or within 5 days after starting systemic treatment, or within 6 weeks from primary diagnosis for patients receiving best supportive care. Patients were excluded if the site of metastasis was not defined. Treatment information was available for every patient. First-line systemic therapy regimens were classified as monotherapy, doublet therapy, triplet therapy, or trastuzumab-containing regimens. Patients who underwent surgical resection (e.g. gastrectomy or surgical resection of metastatic disease) were excluded. This exclusion also applied to patients who received CRS-HIPEC, as this procedure was performed on a very limited scale in selected patients as part of the Dutch PERISCOPE trial.^17^ Patients were categorized based on the anatomic site of the metastases: peritoneum, liver, lung, distant lymph nodes, and other sites (e.g. bone or adrenal gland). Patients were subsequently divided between those with isolated metastases and those with multiple metastatic sites. Patients with metastases at more than one location within the ‘other sites’ category were classified under multiple metastatic sites. For analyses on the effect of systemic therapy (see below), patients with pulmonary metastases were combined with patients with metastases at other sites due to limited group sizes.

### Outcomes

The primary outcome of this study was overall survival (OS). OS was defined as the time from primary diagnosis to death or the end of follow-up, whichever occurred first. Secondary outcomes included differences in patient and tumour characteristics, as well as the association between systemic therapy and survival across different metastatic sites.

### Statistics

OS was calculated using the Kaplan–Meier method for each metastatic site group, and Kaplan–Meier survival curves were compared using the log-rank test. Adjusted multivariable analyses were conducted using Cox-proportional hazards regression models to assess the association between patient, tumour, treatment characteristics, and OS. The hazard ratios (HRs) were presented with 95% confidence intervals (CIs). The multivariable analyses were adjusted for sex, age, baseline Body Mass Index (BMI), comorbidities, World Health Organization (WHO) performance score, HER2 status, Lauren classification, tumour morphology, and administration of systemic therapy. A stepwise backward elimination approach was applied. To investigate the association between the administration of systemic therapy and OS within each metastatic group, HRs with corresponding CIs were calculated by comparing patients who received systemic therapy to those who did not and tested using an interaction term. The Chi-square test for proportions and the Kruskal–Wallis test for continuous data were used to compare baseline characteristics, with distributional assumptions assessed through visual inspection of histograms and Q–Q plots. Two-sided p-values less than 0.05 were considered statistically significant. Analyses were conducted using IBM SPSS Statistics (version 28.0.1.0) and R (version 4.4.0).

### Ethics

As per the Central Committee on Research Involving Human Subjects (CCMO), ethics committee approval is not required for observational, non-interventional studies in the Netherlands. The use of anonymous data for this study was approved by the Privacy Review Board of the NCR (number of approval: K22.188) and informed consent from participants was waived.

### Role of funding source

There was no funding source for this study. The authors had full access to all the data in the study, and the corresponding author had the final responsibility for the decision to submit for publication.

## Results

### Patients

A total of 4272 patients with synchronous metastatic gastric cancer were identified from the NCR database. Of these, four patients were excluded because the metastatic site was not specified, and 196 patients were excluded because they underwent surgical treatment. This included endoscopic resection in 3 patients, gastric resection in 150 patients, and resection of metastases in 68 patients (of which 22 patients underwent CRS-HIPEC). This resulted in a total of 4072 included patients (Supplementary Material: Figure S1). Table 1 shows the patient characteristics.

**Table 1:** Baseline patient characteristics per metastatic site.

Variable	Isolated peritoneal metastases	Non-isolated peritoneal metastases	Non-peritoneal metastases in one organ	Non-peritoneal metastases in multiple organs	Total	p-value
Total, n	1066	769	1408	829	4072	
Sex, n (%)						<0.001^b^
Male	620 (58%)	475 (62%)	1002 (71%)	587 (71%)	2684 (66%)	
Female	446 (42%)	294 (38%)	406 (29%)	242 (29%)	1388 (34%)	
Age, median (IQR), y	71 (61–78)	69 (59–77)	73 (63–80)	70 (61–77)	71 (62–78)	<0.001^a^
Comorbidities, n (%)						<0.001^b^
0	532 (50%)	377 (49%)	594 (42%)	380 (46%)	1883 (46%)	
1	322 (30%)	224 (29%)	431 (31%)	254 (31%)	1231 (30%)	
≥2	163 (15%)	127 (17%)	315 (22%)	151 (18%)	756 (19%)	
Unknown	49 (5%)	41 (5%)	68 (5%)	44 (5%)	202 (5%)	
WHO PS, n (%)						<0.001^b^
0–1	496 (47%)	291 (38%)	592 (42%)	356 (43%)	1735 (43%)	
2	126 (12%)	90 (12%)	168 (12%)	110 (13%)	494 (12%)	
3–4	77 (7%)	84 (11%)	97 (7%)	78 (9%)	336 (8%)	
Unknown	367 (34%)	304 (40%)	551 (39%)	285 (34%)	1507 (37%)	
BMI range, n (%)						<0.001^b^
<20	108 (10%)	56 (7%)	77 (6%)	36 (4%)	277 (7%)	
≥20 –<25	318 (30%)	192 (25%)	344 (24%)	212 (26%)	1066 (26%)	
≥25 –<30	173 (16%)	152 (20%)	284 (20%)	169 (20%)	778 (19%)	
≥30	71 (7%)	70 (9%)	95 (7%)	67 (8%)	303 (7%)	
Unknown	396 (37%)	299 (39%)	608 (43%)	345 (42%)	1648 (41%)	
Tumor location, n (%)						<0.001^b^
GEJ/cardia/fundus	175 (16%)	202 (26%)	619 (44%)	449 (54%)	1445 (36%)	
Corpus	225 (21%)	169 (22%)	209 (15%)	108 (13%)	711 (18%)	
Antrum/pylorus	261 (25%)	156 (20%)	277 (20%)	106 (13%)	800 (20%)	
Curvature/overlapping	347 (33%)	191 (25%)	213 (15%)	101 (12%)	852 (21%)	
Unknown	58 (5%)	51 (7%)	90 (6%)	65 (8%)	264 (7%)	
Lauren classification, n (%)						<0.001^b^
Diffuse type	655 (61%)	303 (39%)	354 (25%)	183 (22%)	1495 (37%)	
Intestinal type	186 (17%)	240 (31%)	533 (38%)	262 (32%)	1221 (30%)	
Mixed	33 (3%)	21 (3%)	36 (3%)	23 (3%)	113 (3%)	
Unknown	192 (18%)	205 (27%)	485 (34%)	361 (44%)	1243 (31%)	
T stadium, n (%)						<0.001^b^
T1-3	585 (55%)	401 (52%)	807 (57%)	441 (53%)	2234 (55%)	
T4	223 (21%)	134 (18%)	162 (11%)	91 (11%)	610 (15%)	
Unknown	258 (24%)	234 (30%)	439 (31%)	297 (36%)	1228 (30%)	
N stadium, n (%)						<0.001^b^
N0	449 (42%)	145 (19%)	268 (19%)	102 (12%)	964 (24%)	
N+	464 (44%)	505 (66%)	991 (70%)	639 (77%)	2599 (64%)	
Unknown	153 (14%)	119 (15%)	149 (11%)	88 (11%)	88 (12%)	
Tumor morphology, n (%)						<0.001^b^
Signet ring cell	162 (15%)	75 (10%)	78 (6%)	37 (5%)	352 (9%)	
Linitis plastica	209 (20%)	82 (11%)	67 (5%)	23 (3%)	381 (9%)	
HER2 status, n (%)						<0.001^b^
Negative	596 (56%)	421 (55%)	655 (47%)	407 (49%)	2079 (51%)	
Positive	55 (5%)	72 (9%)	161 (11%)	119 (14%)	407 (10%)	
Unknown	415 (39%)	276 (36%)	592 (42%)	303 (37%)	1586 (39%)	
Systemic therapy						0.481^b^
No	609 (57%)	466 (61%)	837 (59%)	489 (59%)	2401 (59%)	
Yes	457 (43%)	303 (39%)	571 (41%)	340 (41%)	1671 (41%)	
						<0.001^b^
Doublet (FOLFOX/CAPOX)	312 (69%)	189 (63%)	317 (56%)	176 (52%)	994 (60%)	
Doublet, other	10 (2%)	7 (2%)	28 (5%)	16 (5%)	61 (4%)	
Triplet with anthracyclines	47 (10%)	30 (10%)	64 (11%)	41 (12%)	182 (11%)	
Triplet with docetaxel	31 (7%)	18 (6%)	15 (3%)	4 (1%)	68 (4%)	
Trastuzumab-regimen	22 (5%)	38 (13%)	92 (16%)	78 (23%)	230 (14%)	
Monotherapy	31 (7%)	18 (6%)	43 (8%)	19 (6%)	111 (7%)	

Abbreviations: BMI, Body mass index; CAPOX, capecitabine and oxaliplatin; HER2, human epidermal growth factor receptor 2; IQR, Interquartile range; FOLFOX, 5-fluorouracil and oxaliplatin; GEJ, gastroesophageal junction; WHO PS, World Health Organization performance status.

a Kruskal–Wallis test was used for comparing the four groups.

b Chi-squared test was used for comparing the four groups.

Of the 4072 patients, 1835 patients (45.1%) presented with peritoneal metastases. Of these 1835 patients with synchronous peritoneal metastases, 58.1% patients (26.2% of total) had isolated peritoneal metastases, and 41.9% (18.9% of total) had non-isolated peritoneal metastases. Non-peritoneal metastases were present in 54.9% of patients, which included isolated liver metastases in 33.3% (18.3% of total), isolated distant lymph nodes metastases in 20.5% (11.3% of total), isolated lung metastases in 3.5% (1.9% of total), and isolated other metastatic sites in 5.5% (3.0% of total). The remaining 37.1% (20.4% of total) had non-peritoneal disease in multiple organs. Table 1 presents the distribution of patient characteristics across subgroups defined by the presence of peritoneal metastases. Compared to patients with peritoneal metastases, patients with non-peritoneal metastases were more frequently male (1095 (60%) vs 1589 (71%), p < 0.001). Moreover, patients with non-peritoneal metastases more commonly had a proximal localization of the gastric cancer tumour (1068 (48%) vs 377 (21%), p < 0.001) and intestinal-type histology (795 (36%) vs 426 (23%), p < 0.001). HER2 overexpression was less frequent in patients with peritoneal metastases (127 (7%) vs 280 (13%), p < 0.001), whereas tumours in patients with peritoneal metastases were more likely to be a signet-ring cell gastric carcinoma (237 (13%) vs 115 (5%), p < 0.001). The use of systemic therapy did not differ between patients with peritoneal metastases and those with non-peritoneal metastases (760 (41%) vs 911 (41%), p = 0.481).

### Overall survival

**Fig. 1:**
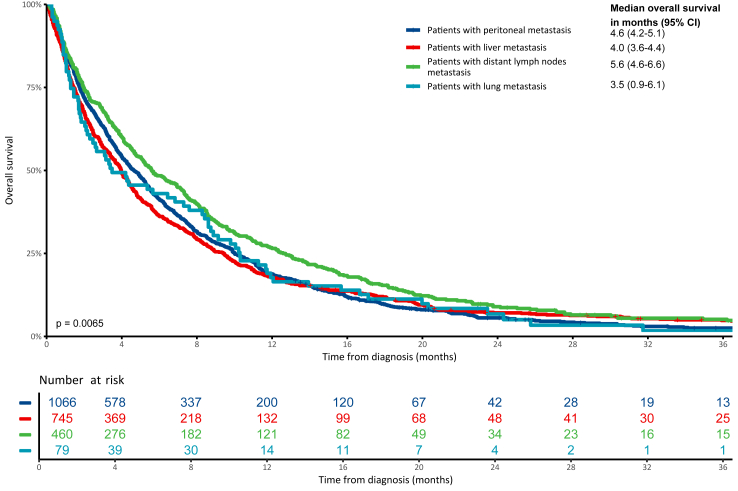
**Overall survival in patients with synchronous metastatic gastric cancer and one metastatic site**. The numbers in the table correspond to the number of patients at risk at each time interval since diagnosis. The p-value represent the result of the log-rank test comparing the Kaplan–Meier survival curves of the different groups. Abbreviation: CI, confidence interval.

The median follow-up of 149 patients without an event for overall survival was 23.9 months (range: 1.8–82.5 months). The median OS for the study population was 3.8 months (95% CI: 3.6–4.0 months). For patients treated with systemic therapy, the median OS was 9.0 months (95% CI: 8.6–9.5 months), while for patients not treated with systemic therapy, who received only palliative care, the median OS was 1.7 months (95% CI: 1.7–1.9 months). The median OS was comparable between patients with isolated peritoneal metastases (4.4 months, 95% CI: 4.0–4.8 months) and those with isolated non-peritoneal metastases (4.6 months, 95% CI: 4.2–5.1 months), with no significant difference in survival between these groups in the multivariable analysis (adjusted HR: 0.94, 95% CI: 0.86–1.03, p = 0.185). Fig. 1 shows the difference in OS between patients with an isolated site of metastasis. Patients with isolated peritoneal metastases (median OS: 4.6 months, 95% CI: 4.2–5.1 months) had a similar survival compared to patients with isolated liver metastases (median OS: 4.0 months, 95% CI: 3.6–4.4 months, adjusted HR: 1.08, 95% CI: 0.97–1.20, p = 0.149) as shown in Table 2 and Table S1. The presence of isolated distant lymph node metastases was associated with a significantly better survival than isolated peritoneal metastases (median OS: 5.6 months, 95% CI: 4.6–6.6 months, vs median OS: 4.6 months, 95% CI: 4.2–5.1 months, adjusted HR: 0.86, 95% CI: 0.77–0.97, p = 0.012). Despite the longer median OS for patients with isolated peritoneal metastases (4.6 months, 95% CI: 4.2–5.1 months) compared to those with isolated lung metastases (3.5 months, 95% CI: 0.9–6.1 months), the presence of isolated lung metastases was associated with significantly better survival in multivariable analysis compared to isolated peritoneal metastases (adjusted HR: 0.63, 95% CI: 0.50–0.80, p < 0.001).

**Table 2:** Overall survival differences among subgroups categorized by site of metastasis.

	Events/total	Median overall survival (months)	Hazard ratio	p-value	Adjusted hazard ratio^a^	Adjusted p-value^a^
**One site of metastasis**						
Peritoneal only	1018/1066	4.6 (4.2–5.1)	Reference	–	Reference	–
Liver only	716/745	4.0 (3.6–4.4)	1.02 (0.93–1.12)	0.682	1.08 (0.97–1.20)	0.149
Distant lymph nodes only	433/460	5.6 (4.6–6.6)	0.84 (0.75–0.94)	0.003	0.86 (0.77–0.97)	0.012
Lung only	76/79	3.5 (0.9–6.1)	1.01 (0.80–1.27)	0.967	0.63 (0.50–0.80)	<0.001
Other isolated site of metastasis	122/124	3.8 (2.3–5.3)	1.06 (0.88–1.28)	0.530	0.97 (0.80–1.18)	0.779
**Peritoneal status**						
Isolated peritoneal metastasis	1018/1066	4.6 (4.2–5.1)	Reference	–	Reference	–
Peritoneal metastasis and at least 1 other site of metastasis	751/769	2.5 (2.2–2.9)	1.38 (1.25–1.51)	<0.001	1.44 (1.31–1.59)	<0.001
Isolated non-peritoneal metastasis	1347/1408	4.4 (4.0–4.8)	0.96 (0.88–1.04)	0.297	0.94 (0.86–1.03)	0.185
Multiple non-peritoneal metastases (≥2 metastatic sites)	807/829	3.1 (2.7–3.5)	1.24 (1.13–1.36)	<0.001	1.38 (1.25–1.53)	<0.001

Abbreviations: BMI, Body mass index; HER2, human epidermal growth factor receptor 2; WHO PS, World Health Organization performance status.

a Adjusted for sex, age, BMI, WHO performance status, comorbidities, HER2 status, Lauren classification, tumour morphology, and use of systemic therapy.

**Fig. 2:**
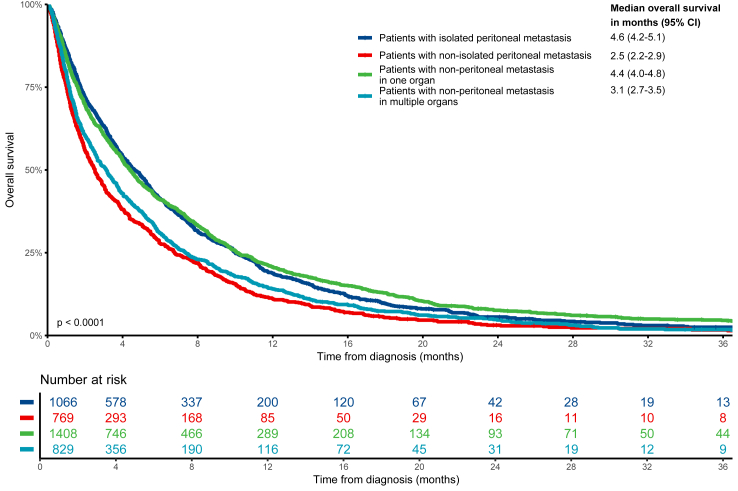
**Overall survival in patients with synchronous metastatic gastric cancer divided by peritoneal status and other metastatic sites**. The numbers in the table correspond to the number of patients at risk at each time interval since diagnosis. The p-value represent the result of the log-rank test comparing the Kaplan–Meier survival curves of the different groups. Abbreviation: CI, confidence interval.

The combination of peritoneal metastases and at least one additional disease site (median OS: 2.5 months, 95% CI: 2.2–2.9 months) resulted in a significantly worse survival than peritoneal metastases only (median OS: 4.6 months, 95% CI: 4.2–5.1 months, adjusted HR: 1.44, 95% CI: 1.31–1.59, p < 0.001, Fig. 2 and Table 2). The presence of multiple non-peritoneal metastatic sites (median OS: 3.1 months, 95% CI: 2.7–3.5 months) was also associated with a worse survival than peritoneal disease only (median OS: 4.6 months, 95% CI: 4.2–5.1 months, adjusted HR: 1.38, 95% CI: 1.25–1.53, p < 0.001). Patients with multiple non-peritoneal metastatic sites (two or more) had similar survival compared to those with peritoneal disease accompanied by metastasis at least one other site (adjusted HR: 0.96, 95% CI: 0.86–1.06, p = 0.394).

### Systemic therapy

The association of systemic therapy on OS for each metastatic site group is presented in Table 3. The survival of patients with isolated peritoneal metastases that received systemic therapy was 10.0 months (95% CI: 9.1–10.9 months), compared to 2.3 months (95% CI: 2.0–2.6 months) for patients with isolated peritoneal metastases that did not receive systemic therapy. A significant association between metastatic site and the effect of systemic therapy was observed (p = 0.041). Surprisingly, the effect of systemic therapy was least pronounced in patients with isolated distant lymph node metastases, where the median OS was 2.9 months (95% CI: 2.2–3.4 months) in patients not treated with systemic therapy compared to 11.2 months (95% CI: 9.5–12.8 months) in patients treated with systemic therapy (adjusted HR: 0.43, 95% CI: 0.33–0.57). Survival was comparable among patients with isolated peritoneal metastases, isolated liver metastases (2.0 months, 95% CI: 1.7–2.3 months, in patients not treated with systemic therapy compared to 9.5 months, 95% CI: 8.5–10.5 months, in patients treated with systemic therapy, adjusted HR: 0.32, 95% CI: 0.26–0.40), and isolated metastases at other sites (2.3 months, 95% CI: 1.7–2.9 months, in patients not treated with systemic therapy compared to 9.3 months, 95% CI: 8.1–10.4 months, in patients treated with systemic therapy, adjusted HR: 0.34, 95% CI: 0.22–0.53) (Table 3). There was no significant difference in overall survival in patients treated with HER2-targeted therapy among subgroups divided by metastatic site (p = 0.136).

**Table 3:** Impact of systemic therapy categorized by site of metastasis.

	Median overall survival (months)		Median survival benefit (months)^a^	Adjusted hazard ratio^b^
**Site of metastasis**	**No systemic therapy**	**Systemic therapy**		
Peritoneal only	2.3 (2.0–2.6)	10.0 (9.1–10.9)	7.7	0.31 (0.27–0.37)
Liver only	2.0 (1.7–2.3)	9.5 (8.5–10.5)	7.5	0.32 (0.26–0.40)
Distant lymph nodes only	2.9 (2.2–3.4)	11.2 (9.5–12.8)	8.3	0.43 (0.33–0.57)
Other isolated site of metastasis	2.3 (1.7–2.9)	9.3 (8.1–10.4)	7.0	0.34 (0.22–0.53)
Multiple sites	1.3 (1.2–1.4)	7.8 (7.1–8.4)	6.5	0.32 (0.28–0.36)

Abbreviations: BMI, Body mass index; HER2, human epidermal growth factor receptor 2; WHO PS, World Health Organization performance status.

a The survival benefit was determined by subtracting the median overall survival of the group without systemic therapy from the median overall survival of the group receiving systemic therapy.

b Adjusted for sex, age, BMI, WHO performance status, comorbidities, HER2 status, Lauren classification, and tumour morphology.

## Discussion

In this study, we investigated the prognostic value of peritoneal metastases in patients with synchronous metastatic gastric cancer in a nationwide, real-world setting. Overall, we did not find a significant difference in survival between patients with isolated peritoneal metastases and those with isolated non-peritoneal metastases. However, patients with isolated peritoneal metastases had significantly shorter survival compared to those with isolated distant lymph nodes or lung metastases, but similar survival compared to those with isolated liver metastases. Moreover, the current data suggest that systemic therapy is equally efficacious for patients with peritoneal metastases compared to those with metastases at other sites in gastric cancer. These outcomes emphasize the heterogeneous nature of peritoneal involvement across different cancers and add to the existing literature by demonstrating that peritoneal metastases in gastric cancer do not confer a distinctly worse prognosis compared to other metastatic sites.

Historically, treatment of peritoneal metastases of gastric origin is associated with limited options and a poor prognosis, as illustrated by the median OS of 4.6 months for isolated peritoneal metastases and 2.5 months for non-isolated peritoneal metastases found in this study. The prognostic value of peritoneal metastases in gastric cancer compared to other metastatic sites is often extrapolated from data on colorectal cancer. However, this study highlights the need for data specifically from patients with peritoneal metastases of gastric origin, rather than extrapolating results from other cancer types such as colorectal cancer. A previous study in colorectal cancer found a pronounced difference in OS between isolated peritoneal and isolated non-peritoneal metastases, with OS of 16.3 months for isolated peritoneal metastases vs 20.0 months for patients with isolated non-peritoneal sites.^11^ This difference was statistically significant, with an adjusted HR of 0.75 (95% CI 0.63–0.91; p = 0.003). In our study, we did not find a significant difference in gastric cancer, with median OS being 4.6 months for isolated peritoneal metastases and 4.4 months for isolated non-peritoneal metastases, and could potentially be explained by differences in pathophysiology.^5^ The mechanisms of peritoneal dissemination are not fully understood, but it is hypothesized to begin with tumour cells shedding from the primary tumour to the peritoneal surface. Other theories suggest that dissemination could also occur through milky spots in the omentum, via lymphatic spread, or even through hematogenous routes.^5^^,^^18^ However, differences in peritoneal transport and adhesion based on the primary tumour type exist, with colorectal cancer more frequently involving multi-cell clusters rather than isolated cancer cells.^19^ Moreover, heterogeneity in the phenotype and genotype of established peritoneal metastases could contribute to differences in prognosis and treatment outcomes. For example, there may be a preferential development of *CMS4* and *BRAF* peritoneal tumours in colorectal cancer, contrasting with the increased frequency of *CDH1* and *TAF1* mutations observed in gastric cancer.^20^, ^21^, ^22^ Differences in prognosis among different tumour types may also be explained by variations in the immune system. A recent study indicated that peritoneal metastases from gastric cancer often feature immature tertiary lymphoid structures associated with immune-suppressive cells like macrophages and regulatory T cells.^23^ This complex tumour immune environment might also contribute to differences in prognostic value, influencing treatment responses and overall survival outcomes.

The current study findings are consistent with known risk factors for synchronous gastric peritoneal metastases in previous literature. These include young age, non-cardia gastric cancer, female sex, signet ring cell carcinoma, T4 staging, and diffuse type histology.^3^ Interestingly, our study also found a significantly lower incidence of HER2 positive disease in patients with peritoneal metastases compared to those without. In a smaller study, authors noted higher HER2 expression in patients with liver metastases compared to those with peritoneal metastases, which led to the speculation that HER2 overexpression may promote liver metastases but not necessarily peritoneal and lymph node metastases.^24^ The exact mechanism behind this discrepancy remains unclear.^25^ Furthermore, our results indicate no significant difference in OS among patients treated with HER2-targeted therapy when divided by metastatic site. However, in view of the small sample sizes within these subgroups, it is important to interpret these findings with caution.

In general, the effect of systemic therapy is considered to be limited in patients with peritoneal metastases, often attributed to the peritoneal-plasma barrier, which results in only a small fraction of systemically administered drugs reaching the peritoneum.^26^ However, in this study, the median OS of patients with isolated peritoneal metastases treated with systemic therapy was 7.7 months longer compared to those who did not receive systemic treatment. This suggests that systemic therapy can be beneficial for some patients with peritoneal metastases, and the difference in OS between treated and non-treated was relatively consistent among patients with isolated peritoneal, liver, and other metastases. This raises the question whether the peritoneal-plasma barrier truly limits the efficacy of systemic therapy, or if other factors have contributed to the observed survival benefit. Although most confounders were included in the multivariable model, selection bias may still exist. For example, no data were available on the volume or burden of disease, and it is possible that patients with a higher peritoneal cancer index (PCI) score, a known prognostic factor, were not selected for systemic therapy.^27^^,^^28^ As a result, the systemically treated patient population may be enriched for those with limited peritoneal metastases, particularly since staging laparoscopy has been part of Dutch guidelines since 2016. Additionally, some patients with low peritoneal disease burden were excluded from this study due to their participation in the PERISCOPE trial. Therefore, while the influence of the peritoneal-plasma barrier on systemic treatment efficacy remains uncertain, our findings indicate that systemic therapy should not be considered universally ineffective for patients with peritoneal disease.

Patients who underwent surgical resection of the primary tumour or metastases were excluded from the analyses, enabling a more homogeneous comparison between the different sites of metastases. This encompassed only a small minority of all patients (68 of 4272), which aligns with current guidelines that do not recommend surgical resection in the metastatic setting.^29^ CRS-HIPEC was applied only on a very limited scale as part of the PERISCOPE-I trial within the excluded population. In the Netherlands, its use is currently under investigation within the PERISCOPE-II trial for patients with limited peritoneal dissemination (i.e., PCI <7) and/or tumour-positive peritoneal cytology.^6^

The field of peritoneal metastases in gastric cancer is rapidly evolving with new treatment strategies continuing to redefine clinical practice. Combining systemic chemotherapy with intraperitoneal chemotherapy, such as catheter-based intraperitoneal chemotherapy and pressurized intraperitoneal aerosol chemotherapy, has the potential to further improve outcomes for patients with peritoneal metastases. Nevertheless, the effectiveness of bidirectional chemotherapy still needs to be validated through ongoing and future clinical trials.^9^^,^^30^ Additionally, the optimal diagnostic modality for peritoneal disease remains under investigation. Techniques such as Magnetic Resonance Imaging (MRI), Fibroblast Activation Protein Inhibitor (FAPI) PET/CT scans, and evaluation of ctDNA in peritoneal fluid hold promise for more accurate diagnosis and evaluation of peritoneal tumour response to therapy. However, a comprehensive understanding of the prognostic implications of peritoneal metastases is crucial for guiding clinical decision-making and optimizing patient care.

To our knowledge, this is the largest study describing outcomes of different sites of metastatic gastric cancer. However, our study has some limitations. First, the NCR does not register detailed information about the extensiveness of the metastatic disease, such as the PCI score or the number of liver metastases. Peritoneal metastatic disease therefore, encompasses the spectrum from positive cytology only to wide-spread macroscopic peritoneal disease. Nevertheless, we believe that this limitation did not substantially impact our results. Notably, a nationwide study conducted in the Netherlands found that overall survival rates were equally unfavourable for gastric cancer patients with positive cytology alone and those with macroscopic peritoneal disease.^31^ Second, due to many missing values for T-stadium, it was not included in the multivariable analysis. Additionally, ethnicity is not collected by the NCR, precluding its inclusion. Another possible limitation is that nivolumab has been registered in the Netherlands for PD-L1 positive metastatic gastric cancer since 2022. As a result, treatment with nivolumab had not yet been introduced in our patient population and data on PD-L1 expression were not available, and the effect of immune therapy on peritoneal metastases could not be studied.

In conclusion, this study shows the prognostic significance of peritoneal metastases in patients with gastric cancer, with no significant difference in survival between patients with isolated peritoneal metastases and those with isolated non-peritoneal metastases. Furthermore, these results indicate that systemic therapy is effective for both patients with peritoneal metastases and those with metastases at other sites in gastric cancer. Interestingly, our findings reveal that the negative prognostic impact of peritoneal metastases compared to other metastatic sites in gastric cancer is less pronounced than expected, highlighting the necessity of disease-specific evaluations rather than extrapolating data across cancer types.

## Contributors

Conceptualisation: NADG, BJN, SLWK, BM, BPLW and RHJM. Data Curation: NADG, MJdN, BJN, HECG, RHAV. Formal Analysis: NADG, BJN. Methodology: NADG, MJdN, BJN, HECG, RHAV, EOdH, BM, BPLW and RHJM. Writing—original draft: NADG and BJN. Writing—review and editing: all authors. All authors read and approved the final version of the manuscript. NADG, MJdN, BJN, HECG, RHAV and RHJM had directly accessed and verified the underlying data in the study.

## Data sharing statement

Data can be made available by the Netherlands Comprehensive Cancer Organization on justified request.

## Declaration of interests

Rob Verhoeven received research grant from BMS, performed consultancy for Daichi Sankyo, all paid to institution. Misha Luyer received consulting fees from Medtronic and Galvani. Ignace de Hingh received unrestricted research grants from Roche and RanD, all paid to institution. Hanneke van Laarhoven received consulting fees from Amphera, Astellas, Beigene, Daiichy, and Myeloid, received research funding, medication supply, and/or other research support from Amgen, AstraZeneca, Auristone, BMS, Incyte, Merck, ORCA, and Servier, and received funding for a speaker role from Astellas, AstraZeneca, BMS, Benecke, Daiichi-Sankyo, JAAP, Medtalks, Novartis, Servier, and Travel Congress Management. Bianca Mostert received consulting fees from Lilly, Amgen, Servier, BMS, AstraZeneca, and research funding from Sanofi, Pfizer and BMS.

The other authors declare that they have no known competing financial interests or personal relationships that could have appeared to influence the work reported in this paper.
