# Supplementary material for: The prognostic value of peritoneal metastases in patients with gastric cancer: a nationwide population-based study
Source: eClinicalMedicine. 2025 Feb 13;81:103109. doi: 10.1016/j.eclinm.2025.103109 (PMC11872448; doi:10.1016/j.eclinm.2025.103109)
Supplement: Supplementary Table and Figure [file mmc1.docx]

**Table of Contents**

[Figure S1: Study flow diagram 2](#_Toc185580066)

[Table S1: Cox regression analyses for overall survival among subgroups categorized by site of metastasis. 3](#_Toc185580067)

# Figure S1: Study flow diagram

**
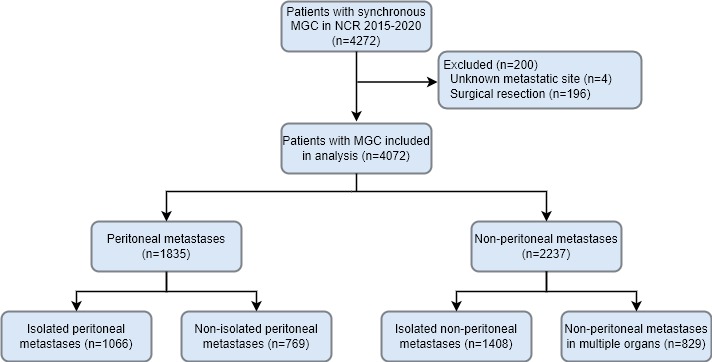
**

*Abbreviations:* MGC: metastatic gastric cancer; NCR: Netherlands Cancer Registry.

# Table S1: Cox regression analyses for overall survival among subgroups categorized by site of metastasis.

|  | Patients, n | Median OS (months) | Univariable analysis | | | Multivariable analysis | | |
| --- | --- | --- | --- | --- | --- | --- | --- | --- |
|  |  |  | **HR** | **95% CI** | **P-value** | **HR** | **95% CI** | **P-value** |
| One site of metastasis | | | | | | | | |
| Peritoneal only | 1066 | 4·6 | Ref |  |  | Ref |  |  |
| Liver only | 745 | 4·0 | 1·02 | 0·93-1·12 | 0·682 | 1·08 | 0·97-1·20 | 0·149 |
| Distant lymph nodes only | 460 | 5·6 | 0·84 | 0·75-0·94 | 0·003 | 0·86 | 0·77-0·97 | 0·012 |
| Lung only | 79 | 3·5 | 1·01 | 0·80-1·27 | 0·967 | 0·63 | 0·50-0·80 | <0·001 |
| Other isolated site of metastasis | 124 | 3·8 | 1·06 | 0·88-1·28 | 0·530 | 0·97 | 0·80-1·18 | 0·779 |
| Sex | | | | | | | | |
| Male | 2684 | 3·8 | Ref |  |  | Ref |  |  |
| Female | 1388 | 3·7 | 1·07 | 0·98-1·14 | 0·060 | 0·95 | 0·89-1·02 | 0·131 |
| Age |  |  | 1·02 | 1·02-1·03 | <0·001 | 1·00 | 0·98-1·00 | 0·702 |
| Comorbidities | | | | | | | | |
| 0 | 1883 | 4·4 | Ref |  |  | Ref |  |  |
| 1 | 1231 | 3·7 | 1·15 | 1·07-1·24 | <0·001 | 1·00 | 0·93-1·08 | 0·990 |
| ≥2 | 756 | 2·6 | 1·48 | 1·35-1·61 | <0·001 | 1·05 | 0·96-1·16 | 0·255 |
| Unknown | 202 | 4·2 | 0·86 | 0·87-1·18 | 0·863 | 0·76 | 0·65-0·89 | <0·001 |
| WHO PS | | | | | | | | |
| 0–1 | 1735 | 6·9 | Ref |  |  | Ref |  |  |
| 2 | 494 | 3·1 | 1·76 | 1·59-1·95 | <0.001 | 1·33 | 1·19-1·47 | <0·001 |
| 3–4 | 336 | 1·4 | 3·37 | 2·99-3·80 | <0·001 | 1·90 | 1·67-2·15 | <0·001 |
| Unknown | 1507 | 1·9 | 1·97 | 1·83-2·11 | <0·001 | 1·46 | 1·35-1·57 | <0·001 |
| BMI range | | | | | | | | |
| <20 | 277 | 3·3 | 1·21 | 1·06-1·39 | 0·005 | 0·97 | 0·84-1·11 | 0.625 |
| ≥20-<25 | 1066 | 4·4 | Ref |  |  | Ref |  |  |
| ≥25-<30 | 778 | 5·2 | 0·92 | 0·83-1·01 | 0·066 | 0·95 | 0·86-1·04 | 0·272 |
| ≥30 | 303 | 4·4 | 0·99 | 0·87-1·13 | 0·098 | 0·91 | 0·80-1·05 | 0·188 |
| Unknown | 1648 | 2·8 | 1·30 | 1·20-1·40 | <0·001 | 1·03 | 0·95-1·12 | 0·476 |
| Lauren classification | | | | | | | | |
| Intestinal type | 1495 | 4·6 | Ref |  |  | Ref |  |  |
| Diffuse type | 1221 | 3·8 | 1·18 | 1·09-1·28 | <0·001 | 1·23 | 0·81-1·86 | 0·341 |
| Mixed | 113 | 5·0 | 1·03 | 0·84-1·26 | 0·769 | 1·54 | 0·86-2·75 | 0·146 |
| Unknown | 1243 | 2·9 | 1·30 | 1·20-1·41 | <0·001 | 1.00 | 0·72-1·38 | 0·983 |
| HER2 status | | | | | | | | |
| Negative | 2079 | 5·3 | Ref |  |  | Ref |  |  |
| Positive | 407 | 7·0 | 0·73 | 0·66-0·82 | <0·001 | 0·78 | 0·69-0·87 | <0·001 |
| Unknown | 1586 | 1·9 | 1·86 | 1·74-1·99 | <0·001 | 1·08 | 0·99-1·17 | 0·052 |
| Tumor morphology | | | | | | | | |
| Signet ring cell | 352 | 3·7 | 0·28 | 0·24-0·34 | <0·001 | 0·46 | 0·31-0·67 | <0·001 |
| Linitis plastica | 381 | 4·3 | 0·28 | 0·23-0·33 | <0·001 | 0·51 | 0·35-0·75 | <0·001 |
| Systemic therapy | | | | | | | | |
| No | 2401 | 1·8 | Ref |  |  | Ref |  |  |
| Yes | 1671 | 9·0 | 0·29 | 0·27-0·31 | <0·001 | 0·34 | 0·31-0·36 | <0·001 |
